# Interprofessional grant writing seminar for early career faculty in a small, isolated teaching center

**DOI:** 10.12688/f1000research.26092.2

**Published:** 2021-07-30

**Authors:** India King, Andrea Christopher, Ann Hansen, Ami Student, Jeff Sordahl, Sarah Naidoo, Elaine Nguyen, Sarai Ambert-Pompey, Amber Fisher, Rick Tivis, C. Scott Smith

**Affiliations:** 1 Department of Psychology, Family Medicine Residency of Idaho, Nampa, ID, 83704, USA; 2 Department of Psychology, Boise VA Medical Center, Boise, ID, 83702, USA; 3 Department of Medicine, Boise VA Medical Center, Boise, ID, 83702, USA; 4 Department of Medicine, University of Washington, Seattle, WA, 98195, USA; 5 Department of Pharmacy, Boise VA Medical Center, Boise, ID, 83702, USA

**Keywords:** Grants, Organized Financing, Mentoring, Peer, Interprofessional

## Abstract

Small, isolated teaching centers have difficulty mentoring interprofessional junior faculty in research methods and grant writing. Peer mentoring programs for grant writing at larger institutions have been successful. In this short report, we describe our program that leveraged mentor experience using four framing seminars followed by project refinement in three-person peer groups and monthly mentored works in progress meetings. In its first year, ten faculty from medicine, psychology, and pharmacy completed the program and successfully obtained six funded grants. Five of the projects transitioned from single profession applications to interprofessional applications as participants connected and profession-specific expertise was identified. Refinements for future cohorts are discussed.

## Introduction

Over the past three decades there has been a decline in research funding ( Alberts *et al*., 2014). The funding that is distributed in many disciplines goes to better established scientists and large, highly networked institutions ( Szell & Sinatra, 2015; Traynor & Rafferty, 1999). Yet, research is an important element for early career faculty promotion in healthcare disciplines ( Yeh *et al*., 2015). One solution that has been successful at larger institutions is self-organized peer mentoring ( Johnson *et al*., 2011). This short report describes a year-long seminar at a small, isolated teaching center designed to expose early-career interprofessional faculty to basic research principles and then to facilitate small group peer mentoring in order that they might obtain their first research awards.

The Boise Veterans Affairs Medical Center (VAMC) provides primary, secondary, and specialty care to over 28,000 veterans each year in an outpatient system that includes five community-based outreach clinics around the state, a 46-bed hospital, an 11-bed inpatient substance abuse treatment center, and a collocated 28-bed nursing home. Boise VAMC has affiliations with the University of Washington School of Medicine (600 miles away), Gonzaga University Nurse Practitioner program (400 miles away), Idaho State University School of Pharmacy (250 miles away) and Boise State University School of Nursing. The facility maintains multiple training programs, including a nurse practitioner residency, pharmacy PGY 1 and PGY 2 residencies, psychology internship and postdoctoral residency, and an internal medicine residency.

## Methods

Our local research office, The Boise VA Office of Research and Development, determined that our outcome data collection would constitute Quality Improvement work and would be considered exempt from IRB approval. The data collected was provided voluntarily by the group participants who gave permission to publish the results in a de-identified format.

Being a small academic facility, far from our major affiliates, and without a deep and rich network of research mentors, we decided to create a grant writing program with the following objectives:

1) Help interprofessional junior clinician educators obtain their first research or program development/evaluation funding.
2) Create a small group of peer-mentors with the experience to recapitulate this effort with future cohorts.

We developed a year-long program that consisted of four kick-off seminars delivered weekly followed by dividing into small project groups of three that collaborated to refine their questions and methods and share the work of information gathering such as grant opportunities, where to find online human subjects forms, etc. Each group reported back to the larger group approximately monthly during ‘works in progress’ (WIP) meetings.

The kick-off seminars were developed by a biostatistician (RT) and a local mentor (CSS) with previous success in obtaining National Institutes of Health, Veterans Affairs (VA), and foundation research funding as well as large program grants. They used a “flipped classroom” format with a research article covering the topic for the session handed out ahead of time to focus and stimulate class discussion and small group interaction. The topics and structure of these seminars are provided in Table 1.

**Table 1.** Description of the four initial seminars involved in the grant writing program.

Seminar Title	Topics	Handouts	Articles for Discussion
The research question	Sharing potential research questions. Discuss research paradigms - Reductionist - Constructivist - Realist	Identifying your research question Summary of research paradigms Research paradigm examples	Wong *et al.* (1) Realist Methods.
Research versus QI & Program Evaluation	Is this research? Example-based discussion pointing out key elements. - Intent to generalize - Design (randomization, double-blind, etc.) - Both can publish!	VHA operations decision tree Squire 2.0 Guidelines (3)	Sanders *et al.* (2) Producing useful evaluations in medical education.
Experimental versus Quasi-experimental designs	When you can’t do an RCT due to cost, ethics, or other constraint, how can you control for confounders?	Table 1 & Table 2. Campbell & Stanley STROBE guidelines (5) Decision tree approach to analytic techniques	VanderWeele & Ding (4) Sensitivity analysis, E-values.
Intro to Qualitative Research	- Overall concepts - Study designs - Sampling strategy - Analytical methods - Adjudication - Software support	Qualitative research summary Data from actual study. Small groups analyze for themes.	Inui (6) The virtue of Qualitative and Quantitative research.

(1) Wong G, *et al.* (2012). Realist methods in medical education research: What are they and how can they contribute. *Medical Education*;46:89–96. (2) Sanders, J., Brown, J., & Walsh, K. (2017). Producing useful evaluations in medical education. *Education for Primary Care, 28*, 137–140. (3) Squire 2.0 guideliens http://squirestatement.org/index.cfm?fuseaction=Page.ViewPage&PageID=471 (4) VanderWeele, T.J., Ding, P. (2017). Sensitivity analysis in observational research: Introducing the E-value. *Annals of Internal Medicine, 167*, 268–274. (5) Strobe guidelines https://www.strobe-statement.org/fileadmin/Strobe/uploads/checklists/ STROBE_checklist_v4_combined.pdf (6) Inui TS (1996) The virtue of qualitative *and* quantitative research. *Ann Intern Med*;125:770–771.

Following these seminars, the participants were divided into groups of three based on rough similarity of research questions and/or proposed methods. These groups decided how often to meet and how to support each other with moving forward on their projects.

Approximately monthly the large group had a WIP update. There was a report on the current state of each three-person group’s projects and any questions or barriers would be outlined. These sessions tended to distribute early expertise. For example, when one member discovered a new funding opportunity it was shared with the entire group. Or, if someone wondered how to navigate the IRB process, someone else who had navigated it would explain where the electronic forms were and offer to help. In addition, an early career faculty member from our affiliate, who had been a successful career development awardee, spoke to the group about the career development program and offered her contact information and assistance.

## Results

Twelve (12) participants started the grant writing program. This included five (5) physicians, four (4) psychologists, and three (3) pharmacists. All but two completed the series (both withdrew from the program due to competing demands). Ten (10) were within three years of being hired to the Boise VA, and ten were academically early career, having entry level faculty appointments. Most had completed programs with less emphasis on research (e.g., PsyD or PharmD versus PhD, fellowships with clinical versus research emphasis).

After completing the grant writing program, six of the remaining ten participants submitted at least one research grant or program evaluation proposal for a total of 10 proposals. Six of these proposals were funded (see Table 2).

**Table 2.** Characteristics of the participants and grants submitted.

Participant (role)	Years since training	Prior grant writing experience	Title of submitted grant	Funding agency (amount awarded if applicable)	Funded
Physician 1 (co-invest.)	26	Y (NIH)	NCI and VA interagency group to accelerate trials enrollment (NAVIGATE)	National Cancer Institute	-
Physician 2 (co-PI)	2	N	Advocacy 101: A curriculum for advocacy for medical trainees in Idaho	Idaho Medical Association Foundation (US$5000)	+
			Advocacy 101: A curriculum for advocacy for medical trainees in Idaho	St. Luke’s Healthcare System, Executive Committee Grant (US$5000)	+
Physician 3 (PI; co-invest.)	2	Y (veterinary)	Improving access for women veterans through training: Opportunity for resource support for women’s musculoskeletal health training programs	VA comprehensive women’s health office of women’s health services (US$57,320)	+
			Education in musculoskeletal care, Innovation network award	Center for Health Professions Education in Musculoskeletal Care (US$46,817)	+
Physician 4	3	N	Did not submit		
Physician 5	2	N	Withdrew from program		
Psychologist 1 (PI)	1	N	Exploring veteran experience of an interprofessional train primary care clinic	VA HSR&D Merit Review, pilot project program	-
Psychologist 2	2	N	Did not submit		
Psychologist 3	2	Y (dance non- profit)	Did not submit		
Psychologist 4	6	N	Withdrew from program		
Pharmacist 1 (PI; co-invest.)	2	N	Impact of statin therapy on influenza virus and vaccine	NIH small grant program (R03)	-
			Community-based influenza screening, testing, and treatment.	Portneuf Health Trust (US$10,275)	+
Pharmacist 2 (PI)	19	N	Facilitating innovative health education scholarship	University of Washington, Center for Leadership and Innovation in Medical Education (US$4000)	+
Pharmacist 3	11	N	Withdrew from program		

Invest. = investigator; PI = principal investigator; NCI = National Cancer Institute; VA = Veterans Affairs; HSR&D = Health Services Research & Development; NIH = National Institutes of Health.

Additionally, we assessed participant satisfaction with the seminar series and continued productivity. Two years after the seminars were conducted we devised a brief impact questionnaire consisting of 5-point Likert-style questions (strongly disagree-1 to strongly agree-5) and a free text area. Response rate was 60%. The results were as follows ( Table 3).

**Table 3.** Results of satisfaction survey.

Question	Score (1 SD to 5 SA)	Standard deviation
Knowledge and skills for design/grant writing	4.5	+/- 0.55
Developed collaborators	4.2	+/- 0.4
Learned about new funding opportunities	4.2	+/- 0.75
Gained confidence	4.2	+/- 0.41
Improved academic career	4.3	+/- 0.52

Comments identified *networking* as the most important element (67% mentioned it) followed by increased confidence, new grant opportunities, and new skills mentioned by one responder each.

This cohort has gone on to submit at least 17 new grants, 6 (35%) of which six were funded for nearly $800,000. Funders included HRSA, VA Office of Academic Affairs, Idaho Dept. of Health and Welfare, and Alaska Dept. of Health & Social Services.

## Discussion

This grant writing seminar and peer support group met the initial objectives of the program and had some unintended positive consequences. The majority of the completing group members submitted a grant application. For most this was their first attempt. Five of the funding applications transitioned from single profession applications to interprofessional applications as connections were made between attendees in their small groups and profession-specific expertise was identified. Because funding sources look for team-based applications from multiple professions ( Wuchty *et al*., 2007), this consequence may have a positive impact on funding success as interprofessional teams at this facility create connections and expand expertise and project success.

Lessons learned include starting the seminar series earlier in the academic year. We started the seminars in mid-summer and this created unnecessary time pressure for proposal submissions, which are usually due in the fall. Also, we would now consider prior experience as a factor as we create the small groups of three.

Our next step was to involve further partnership with the Boise VAMC’s research department. Unfortunately this seminar series was discontinued due to loss of key organizers (no longer working at the site) and a restructuring of the research department.

The research department is interested in expanding the scope and number of research projects and our modest success has demonstrated value. Our research department has agreed to partner in the ongoing development and expansion of this group. This partnership will increase access to instructors from the IRB, VA Health Services Research and Development (HSR&D) office and other major funders of VA research, and the Office of Research Oversight, to discuss distinctions between program evaluation/quality improvement research. These opportunities for direct discussion and questions will be invaluable to the next round of beginning grant writers.

This project had some weaknesses. Because it was not designed as research, no systematic survey of participants (satisfaction, skill improvements, etc.) was obtained. Several topics were identified that might have made the project more effective including types of grants available, navigating the IRB, writing your letter of intent, and budgets. These should be incorporated into future versions.

## Conclusions

Small, isolated teaching centers struggle to provide support for designing and submitting research grants for their early career faculty. Any experienced potential mentors are quickly overwhelmed with mentees. Yet, research is an important element for these same early career faculty’s success. This program of four orienting seminars, three-person project groups, and monthly works in progress meetings was successful in obtaining initial grants for individuals and group expertise that could guide future cohorts. Components of the seminar are site-specific, but we still believe that it is feasible for other sites to adopt and adapt it to meet their individual needs. However, continuity of the *program* appears to depend somewhat on faculty and institutional stability. Individuals perceived a broad range of benefits, most notably creating a network of like-minded researchers. The original cohort continued to write and obtain grants at a high level.

## Data availability

All data underlying the results are available as part of the article and no additional source data are required.
