# Supplementary material for: Interprofessional grant writing seminar for early career faculty in a small, isolated teaching center
Source: F1000Res. 2021 Jul 30;9:1208. Originally published 2020 Oct 8. [Version 2] doi: 10.12688/f1000research.26092.2 (PMC8366299; doi:10.12688/f1000research.26092.2)
Supplement: Peer review history

# Peer review: Reviewer response for version 2

**DOI:** 10.5256/f1000research.30851.r90792

**Published:** 2021-08-13

**Authors:** Mamta Singh

**Affiliations:** 1 VA Northeast Ohio Health Care System, Cleveland, OH, USA; 2 Professor of Medicine, Case Western Reserve University , Cleveland, OH, USA

Thank you for the revisions and comments on sustainability.

 The satisfaction results as well as the success of the initial cohort are helpful and speak to the benefits of this program.

 Thank you for taking the time to present those here.

 I am sorry to hear that the program did not continue as it seems to have been a springboard for interested faculty at your institution.

Is the work clearly and accurately presented and does it cite the current literature?

Yes

If applicable, is the statistical analysis and its interpretation appropriate?

Not applicable

Are all the source data underlying the results available to ensure full reproducibility?

Yes

Is the study design appropriate and is the work technically sound?

Yes

Are the conclusions drawn adequately supported by the results?

Yes

Are sufficient details of methods and analysis provided to allow replication by others?

Yes

Reviewer Expertise:

QI, Patient Safety

I confirm that I have read this submission and believe that I have an appropriate level of expertise to confirm that it is of an acceptable scientific standard.

# Peer review: Reviewer response for version 1

**DOI:** 10.5256/f1000research.28793.r72757

**Published:** 2020-11-17

**Authors:** Dawn DeWitt

**Affiliations:** 1 The Department of Medical Education and Clinical Sciences, The Elson S. Floyd College of Medicine, Washington State University Spokane, Spokane, WA, USA

This is a well-written brief, descriptive report of a capacity-building program at a relatively isolated small academic center.  The detail is enough to replicate the program and although some of the references are relatively old (Inui), they are sound and useful. I would refer others to this article if they were interested in building a similar program.

Is the work clearly and accurately presented and does it cite the current literature?

Yes

If applicable, is the statistical analysis and its interpretation appropriate?

Not applicable

Are all the source data underlying the results available to ensure full reproducibility?

No source data required

Is the study design appropriate and is the work technically sound?

Yes

Are the conclusions drawn adequately supported by the results?

Yes

Are sufficient details of methods and analysis provided to allow replication by others?

Yes

Reviewer Expertise:

Medical education, career choice, diabetes, inter professional education.

I confirm that I have read this submission and believe that I have an appropriate level of expertise to confirm that it is of an acceptable scientific standard.

# Response

**Published:** 2020-11-17

**Authors:** C. Scott Smith

**Affiliations:** University of Washington, USA

Thank you for your time, effort, and suggestions. We will be adding a post-script in response to another reviewer's suggestions you may want to see.

# Peer review: Reviewer response for version 1

**DOI:** 10.5256/f1000research.28793.r72758

**Published:** 2020-11-06

**Authors:** Mamta Singh

**Affiliations:** 1 VA Northeast Ohio Health Care System, Cleveland, OH, USA; 2 Professor of Medicine, Case Western Reserve University , Cleveland, OH, USA

This is a brief report describing a grant writing seminar for early career interprofessional faculty in a small teaching center. The article presents the description of the 4 seminars that were included in this larger grant writing program and the authors give clear descriptions of the participants and their demographics. The article only covers one year and is limited to 10 persons (started with 12- but 2 faculty left the program due to completing demands). Below are some areas that need to be addressed:

1. This is a brief report on a self select group of faculty attending a series of seminars over one year. This narrows the impact of such a program and also speaks to feasibility. Moreover, this poses questions of whether this program could be reproduced at other sites. The authors point out that small isolated teaching centers struggle to provide support for early career faculty and suggest that such a seminar format may be a method to provide support. However,  without evidence that this is sustainable, the report does not help the feasibility argument. If the program is still going on, then comments on how it is being sustained would be helpful.
2. The authors may want to interview the faculty and present some data as to what the faculty thought and how this was helpful. This will provide a "lessons learned" part which again can make the findings helpful to other academic centers in similar situations.
3. It is commendable that the 6/10 programs were funded and is a key outcome for the program. It would be helpful to know if the faculty participants went on to get other funding or seek other funding after completion of the program.

In summary, this is a descriptive report on a grant writing seminar and it is well described and I commend the authors for their work on this. However, in response to their own concern that faculty in isolated centers do not get mentoring in such areas, it would be helpful if they can show evidence of the sustainability of the program, or demonstrate if faculty continue to write grants or bring in funding. 

 As the faculty are self select, if the authors could comment on what worked so other places could reproduce this at their home institutions- it would further the experience for others.

Is the work clearly and accurately presented and does it cite the current literature?

Yes

If applicable, is the statistical analysis and its interpretation appropriate?

Not applicable

Are all the source data underlying the results available to ensure full reproducibility?

Yes

Is the study design appropriate and is the work technically sound?

Yes

Are the conclusions drawn adequately supported by the results?

Yes

Are sufficient details of methods and analysis provided to allow replication by others?

Yes

Reviewer Expertise:

QI, Patient Safety

I confirm that I have read this submission and believe that I have an appropriate level of expertise to confirm that it is of an acceptable scientific standard, however I have significant reservations, as outlined above.

# Response

**Published:** 2020-11-17

**Authors:** C. Scott Smith

**Affiliations:** University of Washington, USA

Thank you for your time and your comments. We agree that components of the seminar are site-specific, but still believe that it is feasible for other sites to adopt and adapt it to meet their individual needs. Regarding *sustainability*, unfortunately this seminar is not still happening due to loss of key organizers (no longer working at the site) and a restructuring of the research department.

*Satisfaction*: We devised a brief impact questionnaire consisting of 5-point Likert-style questions (strongly disagree-1 to strongly agree-5) and a free text area. This was administered two years after the program. Response rate was 60%. The results were as follows:

             Knowledge and skills for design/grant writing          4.4       +/-  0.55

             Developed collaborators                                          4          +/-  0

             Learned about new funding opportunities                4          +/- 0.71

             Gained confidence                                                   4.2       +/- 0.45

             Improved academic career                                      4.2        +/- 0.45

 Comments identified *networking *as the most important element (67% mentioned it) followed by increased confidence, new grant opportunities, and new skills mentioned by one responder each.

*Other Funding: *This cohort has gone on to submit at least 17 new grants, 6 (35%) of which were funded for nearly $800,000. Funders included HRSA, VA Office of Academic Affairs, Idaho Dept. of Health and Welfare, and Alaska Dept. of Health & Social Services.

 As you can see, sustainability of the program was poor, while sustainability of the initial cohorts' grant writing was excellent, largely due to networking, skills, and confidence. We will add these data in a post script.
